# Functionalized graphene oxide NPs as a nanocarrier for drug delivery system in quercetin/ lurbinectedin as dual sensitive therapeutics for A549 lung cancer treatment

**DOI:** 10.1016/j.heliyon.2024.e31212

**Published:** 2024-05-14

**Authors:** Ruomin Liao, Yi Zhang, Wenwei Mao

**Affiliations:** a Department of Respiratory, Shanghai Gerneral Hospital, Shanghai, 201620, China; b Department of Thoracic Surgery, Shanghai Gerneral Hospital, Shanghai, 201620, China; c Department of Respiratory and Critical Care Medicine, The First People's Hospital of Wenling, 317500, Zhejiang Province, China

**Keywords:** Drug, Graphene oxide, Nano carrier, Drug delivery, Lung cancer

## Abstract

Functionalized graphene oxide nanoparticles (NPs) have emerged as promising nanocarriers for drug delivery in lung cancer therapy. Quercetin and lurbinectedin encapsulated in graphene oxide (GO) NPs are tested for treating A549 lung cancer cells. Spectroscopic analyses show that graphene oxide functionalization creates a transparent, smooth surface for drug loading. Treatment with quercetin/lurbinectedin-loaded GO NPs induces notable cytotoxic effects in lung cancer cells, as evidenced by distinct morphological alterations and confirmed apoptotic cellular death observed through fluorescence microscopy. Additionally, our study highlights the impact of this approach on lung cancer metastasis, supported by qRT-PCR analysis of relative gene expression levels, including p53, Bax, Caspase-3, and Bcl 2, revealing robust molecular mechanisms underlying therapeutic efficacy against A549 and PC9 cell lines. Flow cytometric analyses further confirm the induction of cellular death in lung cancer cells following administration of the nanoformulation. Our findings show that quercetin/lurbinectedin-loaded GO NPs may be a promising lung cancer treatment, opening new avenues for targeted and effective therapies.

## Highlights

• Quercetin/Lurbinectedin loaded with graphene oxide NPs (Qn/Ln-GO NPs) sensitive therapeutics for A549 lung cancer treatment.
• RT-PCR relative expression gene p53, Bax, Caspase-3 and Bcl_2_ shows the expression level of A549 and PC9 cell lines.
• Cytotoxicity done with A549 and PC9 cell lines for dual drug, combined drug, and Qn/Ln-GO NPs.
• Flow cytometric analyses also revealed the cellular demise of lung cancer cells.

## 1 Introduction

Among all malignancies, lung cancer has one of the highest fatality rates (American Cancer Society, 2019). When used alone or in conjunction with other treatments like chemotherapy or surgery, radiation treatment (RT) has been crucial in the fight against cancer [1]. Graphene is gaining attention as a potential nanocarrier for drug delivery, joining a growing list of nanocarriers utilized to transport a wide variety of medicinal compounds [2]. Graphene consists of flat, two-dimensional sheets that are only one atom thick. Found in 2004 by Geim et al. [3]. Many scientists are interested in developing nano medicine delivery systems. GO's huge particular surface, substantial drug loading rate, and pH-responsiveness are all excellent features in this respect [4]. Chemotherapeutic resistance to drugs, incorrect diagnosis, poor prognosis are common factors that reduce the efficacy of treatment. Although they are not without their drawbacks, chemotherapy as well as radiotherapy are currently the most successful treatments available. To overcome these obstacles, researchers are focusing on researching the features of the tumor environment and creating new drugs/delivery methods that target over-expressed components [5]. Surface properties of nanoparticles can vary according to factors like surface charge, particle size, presence of either hydrophilic or hydrophobic functional groups, covering materials, and targeting ligands [6]. Anticancer drugs/genes and tests for cancer may be transported more precisely using G and GO-based nanocomposites because to their large surface areas, simplicity of modification, substantial drug-carrying capacity, and induction potential of reactive oxygen species (ROS) [7]. Cell migration is an essential procedure whereby cells need to shift and attain their correct placement in the surroundings in order to perform their activity. Inflammatory and cancer metastasis are only two of the many adverse events that can result from uncontrolled cell migration [8]. Since graphene-based graphene oxide (GO) and lowered graphene oxide (rGO), two samples of carbon nanomaterials, are highly water-dispersible and biocompatible, they could be used to transport chemotherapy medicines [9]. Their utilization in cancer nanomedicine is enabled by their tunability in surface functionalization and large surface area for loading large quantities of hydrophobic anticancer medicines via - interaction. The low pH environment seen in the endosomes following intracellular absorption is optimal for drug release because of their pH-responsive drug release characteristic [10]. However, nanoparticles based on graphene have been shown to be effective photothermal agents in the therapy of cancer. Despite GO's high drug loading and efficient photothermal conversion, it is not yet widely used in chemo photothermal applications [11,12]. Bacterial infections in cancerous tissues further complicate the therapeutic process. The occurrence of infections caused by bacteria in malignant cells, for instance, is an intriguing aspect of lung cancer. Cancer cells are shown to alter the lung microbiota in ways that promote increased inflammation and tumor growth. Gram-negative bacteria, such *Escherichia coli*, were identified as one group of bacteria linked to the development and spread of lung cancer. To avoid the inflammatory response and increase the drug's effectiveness, antibacterial substances could be included in the drug-loaded carriers [13]. Ultrasonication (Farmingdale, NY) was used on the collected precipitate before it was filtered through 25 mm filters in syringes (0.2 μm membrane). Extra alterations were made to the GO in the filtrate [14]. High cytotoxic effects were discovered *In vitro* when reduced-GO constructs with good biocompatibility were tested against A549 malignant cells. However, more research is needed to evaluate their additional medicinal potentials [15]. Increased reactive oxygen species (ROS) production and initiation of lactate dehydrogenase release may result from exposure to those bacterially reduced-GO materials (60 ∼ g ml-1) [16]. Multiple difficult challenges, including adaptability, biologic compatibility, biodegradability, toxicities, interface factorizability, and quenching fluorescence potentials, must be taken into account while building sophisticated G-based nanosystems for cancer diagnosis and treatment [17]. Quercetin's potent anti-inflammatory properties were discovered with its potential uses in malignancy and cardiovascular disease prevention [18]. Several research groups have focused on quercetin because of its promising properties, and now it is being used to treat medical conditions and improve people's diets. However, quercetin's stability and general properties are affected by its treatment during the food and pharmaceutical manufacturing processes [19]. Not only can quercetin content and therapeutic efficacy be negatively impacted by changes in pH, the temperature, oxidation, and degradation, but the reverse is also true [20]. As a result, quercetin's principal limitations are associated with its high medicinal dosage (approximately 500 mg twice a day), limited water solubility, and low bioavailability via the mouth and volatility in a physiological media [21]. Effective regulator of oncogenic transcription lurbinectedin (ZEPZELCA™, Pharma Mar, Madrid, Spain) attaches preferentially to guanines in GC-rich regulatory regions of DNA gene promoters [22]. The medicine suppresses oncogenic transcription and causes tumor cell apoptosis by inhibiting attachment of transcription components to their recognition sequences [23]. By suppressing active transcription in tumor-associated macrophages, lurbinectedin also modifies the composition of the tumor microenvironment. Lurbinectedin administration resulted in a marked and selective reduction in the quantity of circulating monocytes as well as macrophages and vasculature in tumor tissues [24]. Together, quercetin/lurbinectedin and graphene oxide NPs were found to regulate MMP1, CCL2, and CTGF gene expression, physiochemical characteristics, and western blotting. Lung cancer cell proliferation might be efficiently suppressed by Qn/Ln-GO NPs via increasing the level of production of transferrin TFRC. Anti-tumor impact, wound scratch assay, proliferating, apoptosis, and novel approaches to treating lung cancer patients in clinical practice are all bolstered by this research.

## 2 Materials and methods

### 2.1 Preparation of graphene oxide

XG Sciences Inc. supplied the raw ingredients for the graphite platelets, which measured width of 100 μm and thickness ranging from 5 to 15 nm. The GO was made using a twist on the Hummers' method [25]. Add 1 g graphite platelet and 23 ml 98 % H_2_SO_4_ to a 250 ml flask and stir magnetically for 12 h. Slowly add 3 g KMnO_4_ to an ice bath below 20 °C. After 20 min of agitation, the flask had been raised to 35–40 °C and swirled. Heat and stir the solution at 65–80 °C for 45 min until pouring 46 ml of deionized water. Increase solution temperature to 98–105 °C and stir for 30 min. Incubate the solution for 5 min at 35–40 °C with after being cooled at room temperature for 1 h, add 140 ml of water and 10 ml of 10 % H_2_O_2_. Before washing, the solution was spun at 10,000 rpm in a centrifuge. 2–3 times with 5 % HCl. It was repeatedly cleaned with deionized water to neutralize precipitation.

#### 2.1.1 Characterization of Qn/Ln-GO NPs

The samples and spectra were recorded and saved on a Photometer by Bruker Tensor 270 (Burker is a town in New England, USA: Massachusetts) for further use in reaction monitoring and the identification and confirmation of distinct functional groups. The samples were subjected to a thermogravimetric analysis (TGA) using the temperature-controlled apparatus (Linseis, Germany, model TGA PT 1000) operating at a percentage of 10 °C/min from 30 to 900 °C in an environment of nitrogen flowing at a rate of 15 ml/min. The size, surface morphology, as well as elemental composition of Micrographs of the nanosheets were taken using a FESEM and an EDX (FESEM device from the ZEISS corporate, Sigma VP; Oberkochen, Germany). For chemical structure characterization, FTIR spectra were recorded using a Bruker Tensor 270 spectra (Burker, Massachusetts, Yorkshire, USA). After evenly coating all samples with gold dust and placing them on a scanning electron microscope (SEM) stub, the size, interface shape, and chemical analysis of nanosheets were ascertained with the use of an electron beam diffraction (EDX) and field emission scanning electron microscope (FESEM) (ZEISS business, Sigma VP; Oberkochen, Germany).

#### 2.1.2 Drug loading and in-vitro controlled release

Quercetin/Lurbinectedin (Qn/Ln) 20 mg was mixed into synthesized GO and stirred overnight to load it as a medication delivery vehicle. Filtration through 100 KDa filter paper after repeated washing at 9000 rpm for 20 min at room temperature unbound Qn/Ln. Qn/Ln with GO complex was resuspended and kept at 4 °C. Phosphate buffer saline was used for dilution and calibration at pH 7.4. Following is the loading efficiency calculation: LE is the ratio of total Qn/Ln to unbound Qn/Ln, divided by 100 %. The Qn/Ln-loaded GO release rate was measured by processing the extracted material with 50 ml PBS at 37 °C and pH 5.3 and 7.4. The drug content was measured using a UV–visible spectrophotometer after 6 h, when 2 mL of PBS had been used in place of the dialysate [26].

#### 2.1.3 Quantitative reverse transcriptase-polymerase chain reaction (qRT-PCR)

Trizol reagents (Invitrogen, Carlsbad, CA) was used to isolate total RNA from PC9 and A549 cells. The mRNA template was used to synthesis cDNA *In vitro* using the SuperScript 1 III First-Strand Synthesis Kit (Invitrogen) after 1 μg of total RNA was processed with DNase I (Invitrogen). RT-PCR relative expression gene p53, Bax, Caspase-3 and Bcl_2_ shows the expression Actin was used as a check on the system. Agarose gel electrophoresis was used to detect the PCR products, and a gel-based imagery examination machine (Tanon 2500) was used for analysis.

#### 2.1.4 Western blot analysis

After preparing whole-cell extracts using PC9 and A549 cells, 40 g of protein was resolved on a 10 % SDS-PAGE gel. Primary antibodies against were incubated with blots at 4 °C overnight p53 shows the expression (1:500), Bax, (1:2000), Caspase-3 and Bcl_2_ shows the expression (1:5000) following masking with TBST comprising 5 % evaporated milk or 3 % bovine serum albumin (Sigma, St. Louis, MO). Secondary antibody was an IgG coupled with horseradish peroxidase, and it was employed as directed by the manufacturer. Densitometric analysis using Imge J (NIH) was used to quantify the concentration of the group of interest relative to Bcl_2_.

#### 2.1.5 Cell culture

Bruceine D (CAS No. 21499-66-1, ≥98 percent pure) was supplied by the Beijing Sheng Zhong Generic drug Chemical Co., Ltd. in China. The chemical was stored in DMSO at a temperature of −20 °C (Sigma-Aldrich, USA). The PC9 and A549 cell lines were acquired by the Chinese Institute of Biochemistry and Cell Biology. They are routinely able to proliferate in RPMI-1640 media (HyClone, USA). All media were supplemented with 10 % foetal bovine serum (HyClone, USA) (v/v) and 1 % penicillin-streptomycin. The cells were maintained in an incubator at 37 °C with 5 % CO_2_.

#### 2.1.6 *In vitro* cytotoxicity

The MTT assay was used to measure cell survival and physiological metabolism in reaction to the drug loaded GO NPs. The MTT colorimetric assay is a standard technique for measuring cell growth in mammals. Tissue culture polystyrene (TPS) 96-well plates were loaded with varying amounts of free pharmaceuticals and drugs-laden Qn/Ln-GO NPs for cytotoxicity testing at the concentrations of 10, 25, 50, 75, and 100 μg/ml. When the cells had been exposed for the appropriate amount of time, 20 μl aliquots of 5 mg/ml 1 MTT in PBS was added into every well and maintained for 4 h at 37 °C to allow metabolically active cells to reduce the MTT. After 45 s, the material was discarded and reconstituted with 200 μl of DMSO that had been thoroughly pipetted and mixed. The final step involved using a multi-well microplate reader (ELIZA reader, Teknika) to measure the OD.

#### 2.1.7 Cell proliferation assay

The Cell Counting Kit-8 (CCK-8) assay was used to determine the percentage of live cells (Dojindo Molecular, Japan). Five thousand cells were added upon each well on a 96-well plate. Results were examined after incubating 96-well plates at 37 °C with 5 % CO_2_ for 1, 3, and 7 days with or without N-acetyl-l-cysteine (NAC) to avoid ROS generation and introducing Qn/Ln-GO NPs at serial doses of 0, 10, 20, 40, 60, 80, and 150 μg/ml. They used an untreated group as a control. Then, the CCK-8 test was performed out by adding 110 μl identifying agent to individual well, and the 96-well plates were permitted to incubated for an additional 2 h at 37 °C. To prevent any interference with the analytical experiment, the accumulated NPs and cell were kept on the initial plate after 2 h of reaction time, while the testing reagents were transferred to a new 96-well plate. Each well's OD was measured using a reader for micro plates (Spectra Max M5, USA) at a constant wavelength of 450 nm. There were six instances of each treatment.

#### 2.1.8 Assessment of nuclei by means of DAPI staining

DAPI and other cell-permeable nucleic acid staining can be used for nuclear morphology and apoptotic debris analysis in fluorescence microscopy. For nuclei staining, Lung cancer cells were cultivated the desired dosage of medicine while continuously cultivating 2 × 104 cells/well in 6 well-culture plates. After rinsing with PBS and staining with DAPI for 10 min at room temperature in the dark, cell nuclei were observed under fluorescence microscopy (Olympus Optical, Tokyo, Japan) at 40× magnification.

#### 2.1.9 Evaluation of ROS

A ROS assay kit (50101ES01, YEASEN, China) was used to quantify the amount of ROS present. A DCFH-DA solution was added to 100,000 600,000 cells that had been resuspended after their previous medicines and growth media had been removed. The cells were incubated for 0.5–4 h at 37 °C, then centrifuged at 600 g min for 3–4 min at 4 °C. After discarding the supernatant and washing the sediment twice with PBS, the sediment was resuspended with the right volume of PBS. Flow cytometry with an innervation wavelength of 488 nm and production wavelength of 525 nm was used to measure ROS levels upon resuspension.

#### 2.1.10 Scratch wound healing migration assay

A scratches wound healing assay was used to measure cell migration. After 24 h of cultivation in six-well plates, PC9 and A549 cells were treated with 50 μM Qn/Ln-GO NPs for an additional 24 h. The cells were resuspended, and 2 × 105 were reseeded into the six-well plates, where they were grown to monolayers and subsequently injured with sterile 1 mL pipette tips. PBS was used to clean the cells and eliminate any remaining debris. Photographs were taken at 0, and 24 h post-injury. Software like ImageJ (National Institutes of Health) can be used to objectively measure the gap distance.

(1) The equations for relative wound area … … … … … … … …

(2) Percentage (%) of wound closure … … … … … … … … … … … … … … … … …

(3) Wound healing speed … … … … … … … … … … … … … … … … … … … … … …

(4) Relative wound area = Wt/W0

(5) Wound closure (%) = ((W0 − Wt)/W0) × 100

(6) Healing speed (μm2/ h) = (W0 − Wt)/ΔT

W0 = Wound area at 0 h (μm2)

Wt = Wound area at Δh (μm2) ΔT = Duration of wound measured (h) [27].

### 2.2 Statistical analysis

To analyse the result of at least 3 different studies, we used the mean standard deviation. For statistical analysis, we used the student's t-test. Any p-value below 0.05 (from a one-way ANOVA or a two-tail student's t-test) was considered to indicate the presence of a statistically significant difference. The GraphPad Prism 8.0 software was used to create the graphs.

## 3 Result and discussion

### 3.1 Characterization of Qn/Ln-GO NPs

**Fig. 1:**
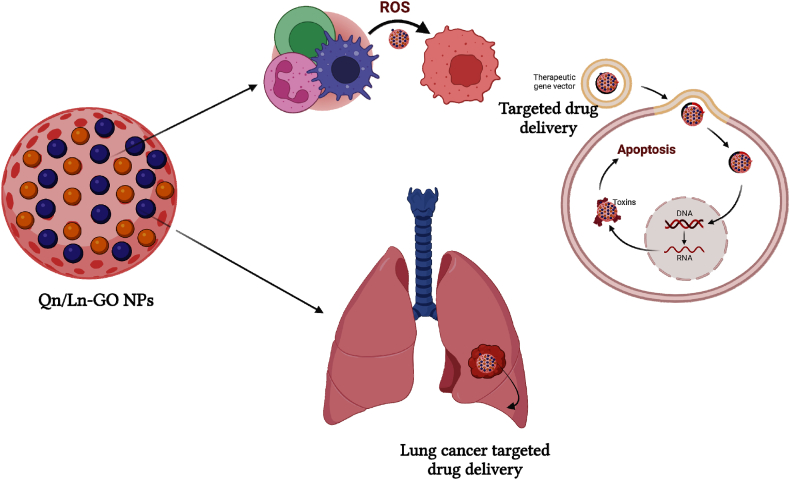
Schematic representation of Qn/Ln-GO NPs and its applications.

**Fig. 2:**
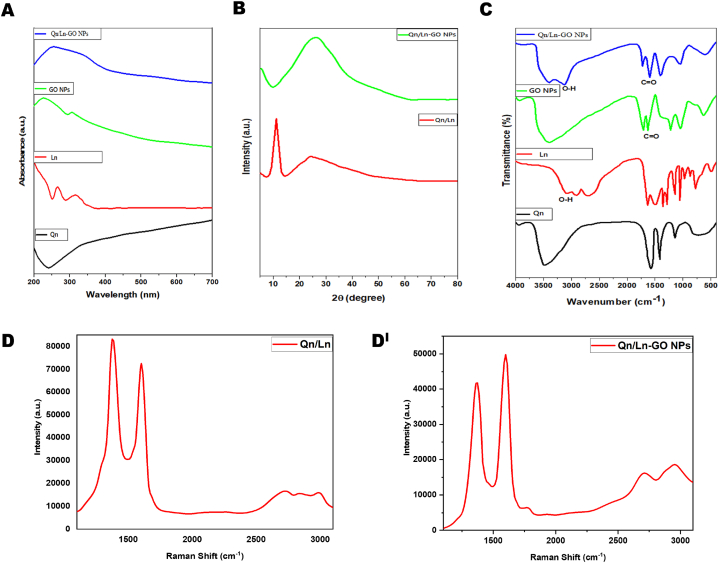
UV − visible spectroscopic analysis of Qn, Ln, GO NPs and Qn/Ln-GO NPs (A), XRD analysis of Qn/Ln and Qn/Ln-GO NPs (B), FT-IR vibrational transmittance of Qn, Ln, GO NPs and Qn/Ln-GO NPs (C), Raman spectroscopic measurements of Qn/Ln and Qn/Ln-GO NPs (D & D^1^).

**Fig. 3:**
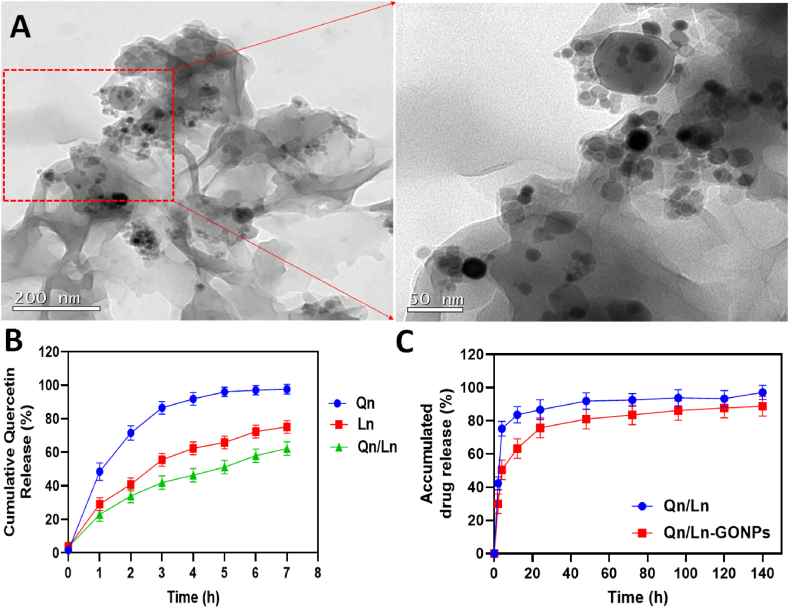
TEM images of Qn/Ln-Zo NPs at 50, 100, 200 nm and 1 mm respectively (A); Cumulative drug Qn release to GO and combined drug Qn/Ln released to GO at different period of time at pH 7.4 (B & C).

In present study, Qn/Ln-GO NPs possess characterization and biomedical application shown as schematic representation in Fig. 1. According to UV–vis spectra, pure Au NPs exhibit a plasmon absorption peak at 520 nm. The core-shell formation of Au@PB NPs was obtained when a PB film was swathed around the surface of Au NPs. Among them, PB peaked around 700 nm [28]. In present study UV − visible spectroscopic analysis of Qn, Ln, GO NPs and Qn/Ln-GO NPs at 250–360 nm (Fig. 2: (A)). Peaks at 2 value systems of 12.97, 14.79, 16.50, 18.32, 19.18, 20.46, 22.50, 23.25, 24.96, 26.35, 29.99, 30.95, 32.98, and 38.44 were visible in an XRD analysis of free Dox. Peaks associated with free Dox disappeared from GO and graphite XRD patterns in the powder form drug-loaded nanoformulations [29]. XRD analysis of Qn/Ln and Qn/Ln-GO NPs (Fig. 2: B). The hybridised nanocomposites, which contained both GO and Hes, were examined using Fourier transform infrared spectroscopy. With a wide medium intensity peak at 3449 cm^−1^, the anticancer drug hesperetin displayed the O–H stretching vibrations of alcohol. At 1013 cm^−1^, the C–F stretching was detected. The GO FT-IR spectrum displayed an alcohol O–H stretching vibration at 3376 cm^−1^, which was broad and strong. The participation of carbon dioxide was demonstrated by a peak at 2363 cm^−1^. Indicating the carbonyl carbon (C=O), the peak at 1633 cm^−1^ was observed [30]. FT-IR vibrational transmittance of Qn, Ln, GO NPs and Qn/Ln-GO NPs (Fig. 2: C). The graphene Raman spectrum is divided into two primary regions, named D-band and G-band. The D-band is at 1350 cm^−1^ and the G-band is at 1575 cm^−1^, respectively, and is caused by the A1g carbon atom. Likewise, in comparison to the graphitic G-band, the Raman spectra of GO showed a wide G-band that was blue-shifted to 1612 cm^−1^, indicating the presence of inaccessible high occurrence binary bonds [31]. Furthermore, the diminished size of sp2 domain oxidation is indicated by the heightened D-band at 1364 cm^− 1^. It is possible that the creation of new small sp2 domains during deoxygenation is responsible for the increase in the force proportion of the D-band to the G-band, which went from 1.43 to 1.50 subsequently decrease. Raman spectroscopic measurements of Qn/Ln and Qn/Ln-GO NPs showed in Fig. 2: (D & D^1^). Photos taken by TEM of the GO/GCE and THi–CS–GO/GCE. The morphology of GO/GCE nanosheets is characterised by the usual thin wrinkled GO morphology, which is associated with a high surface area, permeability, and surface roughness [32]. This same inverted pendulum test and TEM investigations adequately justified the continuity of the calfthymus DNA predicated biomolecular hydrogel material. The TEM-morphological study was the only method used to image the supramolecular hydrogel network based on DNA and 2D graphene nanosheets [33]. TEM images of Qn/Ln-Zo NPs at 50, 100, 200 nm respectively (Fig. 3: A); The morphological features of GO and rGO. The TEM micrograph of GO Several strands have been stacked on top of one another, resulting in the formation of thick, bulky clusters of opaque sheets [15].

#### 3.1.1 Drug loading efficiency

In order to assess the liberation of quercetin from the nanoparticles laden with quercetin, *In vitro* release assays were conducted utilizing the dialysis cellulose diffusion method. In light of the objective of this research, which was to develop fucoidan/chitosan nanoparticles intended for oral administration, the release of quercetin was assessed in a simulated gastrointestinal environment [21]. Several hours of incubation revealed that the drug release from a graphene-oxide and Quercetin/Lurbinectedin as a responsive drug (Qn/Ln-GO NPs) route of administration was pH dependent (Fig: 3 B &C). The presence of a peak at 365 nm and an intensified peak at 280 nm, which correspond to the FA maximum of GO-PEG-FA, in the UV visible spectra analysis, provide confirmation that CPT is loaded onto GO-PEG-FA. Additionally, a loading efficiency of 45 % was determined [26]. The effectiveness of nanoencapsulation is crucial to NP medication delivery. Results showed that Sil had a nanoencapsulation efficiency of 67.4 ± 3.5 %, Met had an efficiency of 80.35 %, and Sil/Met had an efficiency of 82.49 %. In addition, Sil and Met were shown to have a drug lading volume of 13.6 ± 3.1 % and 12.3 ± 4.2 % respectively. It appears that the hydrophobic character of these natural anticancer medicines is responsible for their remarkable encapsulation efficacy [34].

#### 3.1.2 PCR and western blotting analysis

**Fig. 4:**
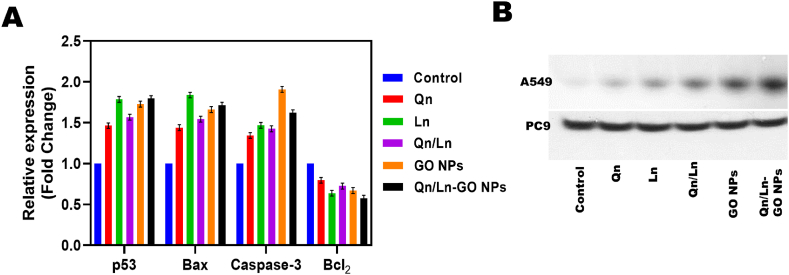
RT-PCR relative expression gene p53, Bax, Caspase-3 and Bcl_2_ shows the expression level of A549 and PC9 cell lines; the bar graph represents the significance difference between groups (A). Western blot shows that PC9 and A549 cells exhibited p53, Bax, Caspase-3 and Bcl_2_ gene expression serves the loading control in Western blot experiments.

The number of copies TFRC was higher in the HH group, as evidenced by the qPCR and IHC results. Based on the finding that lung cancer patients' TFRC expression levels are significantly higher than those of healthy individuals', we anticipated that HH would be able to suppress lung cancer cell growth by increasing TFRC expression [35]. RT-PCR relative expression gene p53, Bax, Caspase-3 and Bcl_2_ shows the expression level of A549 and PC9 cell lines. The Western blot shows that PC9 and A549 cells exhibited p53, Bax, Caspase-3 and Bcl_2_ gene expression aids the lading regulator in Western blot tests (Fig. 4 A&B). Similarly, several cell lines, particularly A549, H1299, H460, SK-MES-1, and H727, displayed explicit migration towards the bottom compartment. Collagen type IV is cleaved by MMP-2, and this cleavage contributes to invasiveness and tumor spread across tissues. Osteolytic metastasis involves the osteoclastogenic factors macrophage incendiary protein (MIP-1-a) and parathyroid hormone-related amino acids (PTHrP). Galectin 3 (LGALS3) enhances metastasis by acting on location of trans endothelial mediators [36]. Metastasis is characterized by a rapid growth rate of cancer cells. Our results show that PRAME act a significant part in lung cancer migration and that reducing PRAME expression significantly increases lung cancer cell proliferation in PC9 and A549. This finding suggests PRAME may have a preventative effect on lung cancer [37]. Cav-1 (Caveolin-1) By demonstrating that Cav-1 transcript is greater in BM compared to the primary carrier of the SQC type, we offer crucial perception hooked on the potential involvement of Cav-1 in EMT through snail [38]. The powerful migratory inhibitory effect of phycocyanin on cancer cells has attracted a lot of interest. Phycocyanin may inhibit melanoma and breast cancer cell migration by acting on MAPK signaling, according to certain reports. Phycocyanin inhibited migration of several NSCLC cells in a first-of-its-kind manner via controlling matrix metalloproteinases 2 (MMP2) and 9 [39]. Furthermore, in order to confirm the specificity of these six proteins, we further investigated each one using an epitope-specific antibody. The results demonstrated that, in contrast to the surrounding normal tissues, the tumor tissues of lung adenocarcinoma had an overexpression of the TAL2 and ILF3 proteins. The oncogenic proteins TAL2 and ILF3 are related [40]. By RT-PCR and western blotting, H1975 has been found to express Wnt3a. We employed real-time PCR analysis to quantify levels of Wnt3a gene expression in order to ascertain whether Klotho-S may impact this level of expression. In comparison to the vector transfection group, A549 and H1975 cells transfected with KLotho-S had lower levels of Wnt3α mRNA [41]. Similarly, RT-PCR was performed on cells that were exposed to A23187 doses ranging from 0 to 6 ÌM for a duration of 24 h. RT-PCR detection of GRP78 mRNA increased concentration-dependently after A23187 treatment; this increase ranged from 2.2- to 4.8-fold relative to untreated cells. Using Western blotting, the expression of GRP78 protein in NCI–H460 cells exposed to varying doses of A23187 (0, 1, 2, 4, 6 ÌM) for 24 h was assessed [42].

#### 3.1.3 Cytotoxicity effects and DAPI staining of Qn/Ln-GO NP

**Fig. 5:**
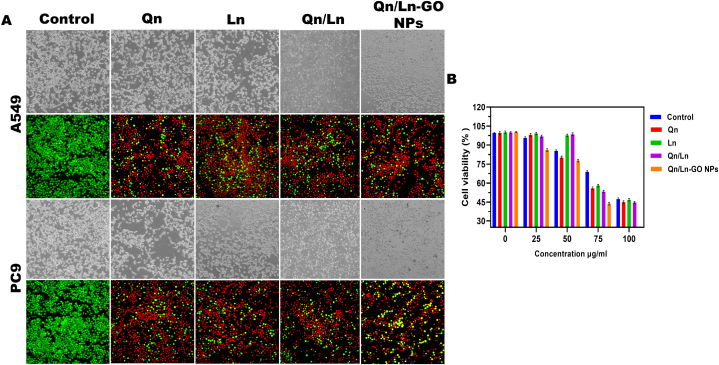
In vitro morphological observations of A549 and PC9 cells treated with Qn, Ln, combined Qn/Ln, and Qn/Ln-Zo NPs at 50 μg/ml. In cancerous cell when the Qn/Ln-Zo NPs treated the cell structure got anomalous and morphologically changed when compared with other groups; Phase contrast and live and dead cell viability at 100 nm (A); Cell viability percentage are significant with p < 0.01 in A549 and PC9 treated with Qn, Ln, combined Qn/Ln, and Qn/Ln-Zo NPs (B).

Electrode surface electron transport has been increased by a factor of six thanks to the addition of an ad layer of AuNPs/rGO. This research paves the way for us to use the PDA/AuNPs/rGO/FTO electrochemical cytosensor already in development to identify A-549 cells. As-prepared, the sensor has a detection limit of 1 cell/ml [43]. *In vitro* morphological observations of A549 and PC9 cells treated with Qn, Ln, combined Qn/Ln, and Qn/Ln-Zo NPs at 50 μg/ml. In cancerous cell when the Qn/Ln-Zo NPs treated the cell structure got anomalous and morphologically changed when compared with other groups; Phase contrast and live and dead cell viability at 100 nm. Cell viability percentage are significant with p < 0.01 in A549 and PC9 treated with Qn, Ln, combined Qn/Ln, and Qn/Ln-Zo NPs (Fig. 5: A&B) The mucoadhesive characteristics of the produced particles enhanced their absorption when taken Phenotypic evaluations of A549 and PC9 cells in a controlled laboratory setting after exposure in the A549 lung cancer cell model, the produced methotrexate delivery systems suppressed cell proliferation by a factor of seven compared to the free drug [44]. When quercetin was added to A549 cells, Akt activation was reduced. Snail upregulation and quercetin-mediated invasiveness inhibition were both abolished in A549 cells through overexpression of activated Akt. These findings point to quercetin's inhibition of Akt as a possible contributor to the inhibition of Snail-induced cell motility [19]. In accordance with the outcomes of a single-arm phase 2 basket trial, lurbinectedin monotherapy is presently licensed for the conduct of adults with advanced small cell lung cancer (SCLC) who have seen progression of the cancer during or following platinum-based chemotherapy. In order to compare the trial's findings of lurbinectedin's effectiveness, especially with regards to the overall survival (OS) and the

**Fig. 6:**
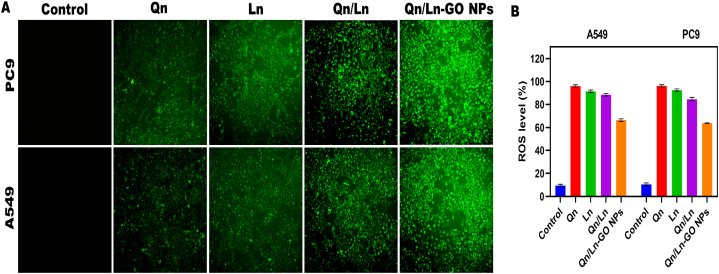
A549 and PC9 cells were subjected to oxidative stress when exposed to Qn, Ln, combined Qn/Ln, and Qn/Ln-Zo NPs. (A) The percentage increase or decrease in reactive oxygen species (ROS) production in A549 and PC9 cells (A); ROS production levels were statistically analysed in A549 and PC9 cells following a 24-h exposure OLYMPUS CKX 41 fluorescent microscope images were used (B).

**Fig. 7:**
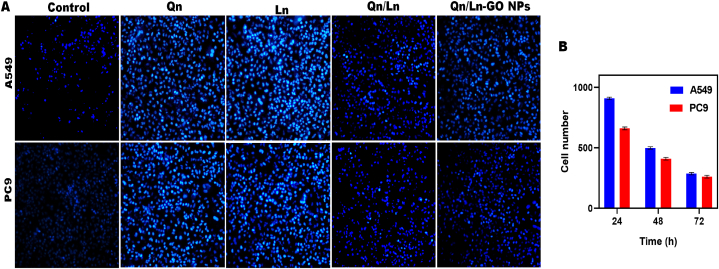
DAPI staining for Blue fluorescent dye was used to label the nuclear DNA of proliferating cells. Qn, Ln, combined Qn/Ln, and Qn/Ln-Zo NPs -treated cells showed cell proliferation when compared to control. The presence of high levels of blue fluorescence and apparent nuclear fragmentation in treated cells is indicative of chromatin condensation (A). Cell proliferation for A549 and PC9 with various periods of time was examined with significant variations p < 0.01 (B). (For interpretation of the references to color in this figure legend, the reader is referred to the Web version of this article.)

Overall response rate (ORR), the current study set up real world comparator arms (RWCAs) [45]. A fusion protein's oncogenic features will always be at odds with those of the wild-type protein it fused with. Relocating EWS-FLI1 inside the nucleus to silence its activity is made possible by activating the double-stranded DNA repair (DDR) of EWSR1 [24]. Both types of lung cancer cells had their proliferation slowed by HH in a dose-dependent fashion. The IC50 of HH for A549 and LLC cells was 9.315 mg/ml and 5.886 mg/ml, accordingly. These findings corroborate one another in demonstrating that HH reduced the proliferation of lung cancer cells. Constant reactive oxygen species (ROS) production and buildup in cells, brought on by the increased creation of hydroxyl radicals, led to cellular malfunction and death [35]. Qn, Ln, combined Qn/Ln, and Qn/Ln-Zo NPs all caused oxidative stress in A549 and PC9 cells. (A) The change in ROS production per unit of time between A549 and PC9 cells. After being exposed for 24 h, the levels of reactive oxygen species (ROS) were statistically examined in A549 and PC9 cells (Fig. 6 A & B). As like, a complete description covering all the characteristics, from the basics to the synthesis and uses, of graphene oxide related nanomaterials is of tremendous value, as current studies frequently concentrate on restricted aspects of these materials [46]. At the molecular, cellular, and organelle levels, oxidative stress is caused by ROS. Different parameters including type of cell, chemical makeup of the surface, shape, size, treatment period, and dose play a vital influence in the action of nanomaterials on cells. By increasing ROS production, which occurs non-enzymatically in the mitochondria, namely in the mitochondrion electron transfer complexes III and I, nanoparticles of silver can activate the components involved in the cell death process [47]. For damaged cells to develop into cancerous tumors, apoptosis may be seen as a significant barrier. Apoptosis (10–13 %) was clearly triggered in A549 cell lines by incubation by allowed GEM, G, GO, GEM-GO, and GEM-GO combined NIR. Compared to the published graphene nanoparticles, the nanomaterials put to the test exhibited significantly higher apoptotic induction [31]. To label the nuclear DNA of cells that were dividing, scientists utilized DAPI staining for blue fluorescent dye. When compared to the control group, cells treated with Qn, Ln, combined Qn/Ln, or Qn/Ln-Zo NPs proliferated. Chromatin condensation is confirmed by the presence of nuclear fragmentation and high rates of blue fluorescence in treated cells. The examination of cell proliferation for A549 and PC9 over different time periods revealed significant variations (Fig. 7: (A &B)).

#### 3.1.4 Apoptosis through flow cytometry analysis

**Fig. 8:**
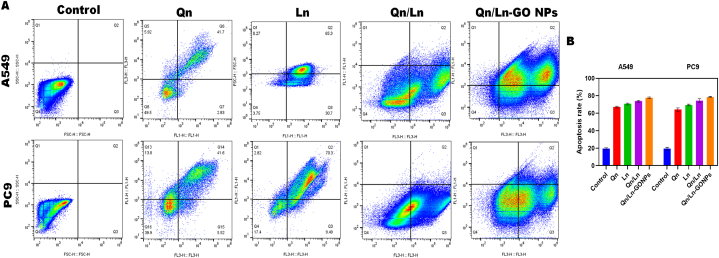
Flow cytometry was used to determine the apoptosis rate. Treatment of A549 and PC9 cells were treated with Qn, Ln, combined Qn/Ln, and Qn/Ln-Zo NPs undergoing cell death (A). The apoptosis percentage were analysed for A549 and PC9 with statistically analysed (B).

It is possible that apoptosis is a major barrier for damaged cells to develop into cancerous tumors. Flow cytometry has shown that nanomaterials can induce cell death in cancer cells. Notably, GEM-GO and GEM-GO combined NIR directly damage even at absorptions lower than their IC50, which is quite low. Annexin V (+)/PI(−) (upper left) quadrant had a higher cell population (6–9 percent) compared to the control, suggesting the sensitization of early apoptosis. The effects were determined to be more pronounced for GEM-rGO and GEMrGO + NIR compared to the free GEM, G, and GO samples, according to the results of the MTT and AO-EB staining investigations. It should be mentioned that the test nanomaterials demonstrated superior apoptotic induction compared to the graphene nanomaterials that were reported [31]. Flow cytometry was employed to ascertain the apoptosis rate in this investigation. Before cell death occurred, A549 and PC9 cells were exposed to Qn, Ln, combined Qn/Ln, and Qn/Ln-Zo NPs. A549 and PC9 had their apoptosis percentages statistically examined (Fig. 8 (A&B).

#### 3.1.5 Wound scratch assay

**Fig. 9:**
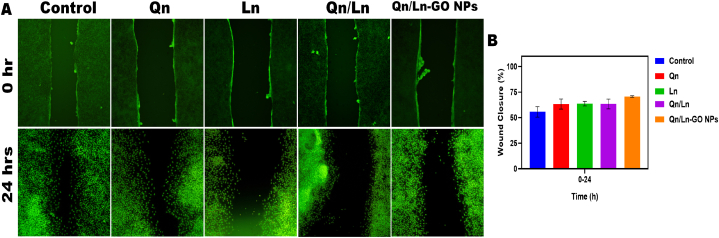
The scratch assay to investigate the wound healing of A549 and PC9 cell lines indicate the area of the scratch at 0, 24, and 48 h after the treatment. Fluorescent Pictures shows the groups treated with Qn, Ln, combined Qn/Ln, and Qn/Ln-Zo NPs with the better healing rate than control (A). Scratch wound area was measured 0,24 and 48 h post treatment. P < 0.001 statistically significance.

Naturally, cells have inclination to undertake migration which is a crucial step for development and to guarantee proper tissue functioning. Cell migration is a key factor in invasion and metastasis by cancer cells. The cancer cells' inevitable demise could be hastened by their adherence, which blocks nourishment from entering the cell. This knowledge is crucial for the development of entirely novel natural C-dots that may one day be used to cure cancer [48]. When these results were compiled, they showed that persistent *In vitro* exposure to cisplatin created a cisplatin-resistant phenotypes in four OC sublines, which in turn triggered metastatic signals characterized by increased cell migratory potential. At 12 h, CisR sublines demonstrated accelerated wound healing compared to parental cells. In OV-90/CisR1, the average wound healing rate was faster in the CisR sublines 36,852.04 ± 1358.73 μm2/h than in the control group 14,339.6291 ± 2901.75 μm2/h [27]. The area of the scratch at 0 and 12 h after treatment is shown in the scratch assay, which investigates the healing process of A549 and PC9 cell lines. The groups that were managed with Qn, Ln, cumulative Qn/Ln, and Qn/Ln-Zo NPs had a superior healing rate than the control group, as shown by the fluorescent pictures. At 0 and 24 h after treatment, the area of the scratch wound was measured (Fig. 9 (A&B).

## 4 Conclusion

Various spectroscopical examinations and characterizations employing various analytical and spectroscopical methodologies supported the smooth and clear morphological surface of the oxide graphene loaded combination drug quercetin/Lurbinectedin in this study. RT-PCR research revealed the expression of Qn/Ln-GO NPs, the majority of which are associated with RT-PCR relative expression gene p53, Bax, Caspase-3 and Bcl_2_ shows the expression level of A549 and PC9 cell lines. In a consequence, Qn/Ln-GO NPs were reported to possess significant *In vitro* cytotoxicity towards the lung cancer cells. Biochemical assays were used to analyse the structural alterations in lung malignance cells; these included AO-EB, fluorescent, phase contrast, and DAPI labelling. Proliferation, apoptosis, and the wound scratch assay can all be predicted with the use of Qn/Ln-GO NPs. Further research into the *In vivo* function of graphene nanoparticles can be informed by the findings reported here.

## Funding statement

This study was supported by the Wenling Social Development Technology Project (NO.2022S00121).

## Data availability statement

Data included in article/supplementary material/referenced in article.

## Additional information

No additional information is available for this paper.

## CRediT authorship contribution statement

**Ruomin Liao:** Writing – original draft, Software, Methodology, Data curation. **Yi Zhang:** Software, Resources, Investigation, Formal analysis. **Wenwei Mao:** Writing – review & editing, Supervision, Project administration.

## Declaration of competing interest

The authors declare the following financial interests/personal relationships which may be considered as potential competing interests: There are no conflicts of interest for the present research work. If there are other authors, they declare that they have no known competing financial interests or personal relationships that could have appeared to influence the work reported in this paper.
