# Supplementary material for: Functionalized graphene oxide NPs as a nanocarrier for drug delivery system in quercetin/ lurbinectedin as dual sensitive therapeutics for A549 lung cancer treatment
Source: Heliyon. 2024 May 14;10(11):e31212. doi: 10.1016/j.heliyon.2024.e31212 (PMC11152904; doi:10.1016/j.heliyon.2024.e31212)

Supplementary Figure 1: Western blot shows that A549 cells exhibited p53, Bax, Caspase-3 and Bcl_2_ gene expression


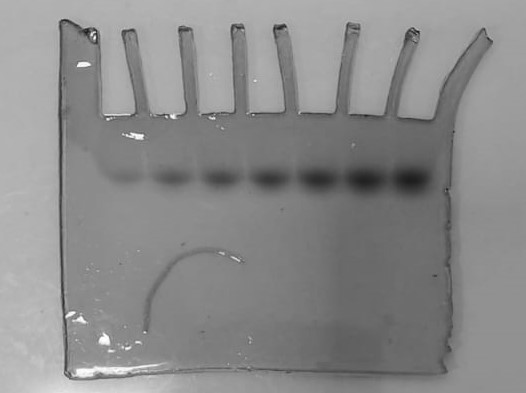


Supplementary Figure 2: Western blot shows that PC9 cells in western blot experiments.


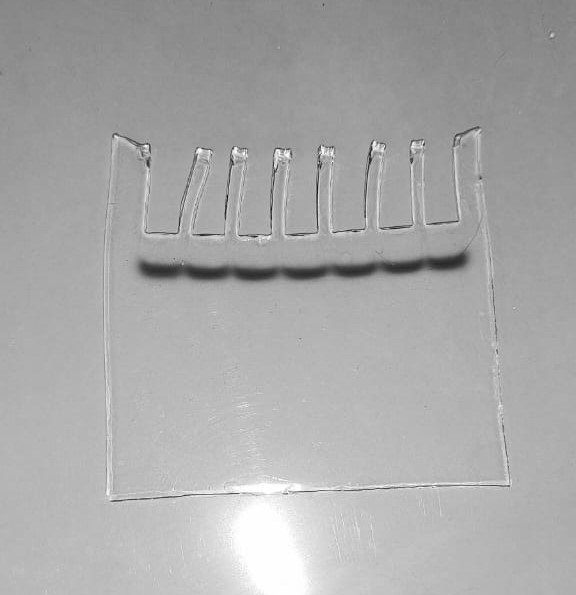

Supplement: Multimedia component 1 [file mmc1.doc]
